# Physically interacting humans regulate muscle coactivation to improve visuo-haptic perception

**DOI:** 10.1152/jn.00420.2022

**Published:** 2023-01-18

**Authors:** Hendrik Börner, Gerolamo Carboni, Xiaoxiao Cheng, Atsushi Takagi, Sandra Hirche, Satoshi Endo, Etienne Burdet

**Affiliations:** ^1^Electrical and Computer Engineering Department, Technical University of Munich, Munich, Germany; ^2^Department of Bioengineering, Imperial College of Science, Technology and Medicine, London, United Kingdom; ^3^NTT Communication Science Laboratories, Atsugi, Kanagawa, Japan

**Keywords:** computational model, electromyography, human-human interaction, muscle coactivation, visuo-haptic perception

## Abstract

When moving a piano or dancing tango with a partner, how should I control my arm muscles to sense their movements and follow or guide them smoothly? Here we observe how physically connected pairs tracking a moving target with the arm modify muscle coactivation with their visual acuity and the partner’s performance. They coactivate muscles to stiffen the arm when the partner’s performance is worse and relax with blurry visual feedback. Computational modeling shows that this adaptive sensing property cannot be explained by the minimization of movement error hypothesis that has previously explained adaptation in dynamic environments. Instead, individuals skillfully control the stiffness to guide the arm toward the planned motion while minimizing effort and extracting useful information from the partner’s movement. The central nervous system regulates muscle activation to guide motion with accurate task information from vision and haptics while minimizing the metabolic cost. As a consequence, the partner with the most accurate target information leads the movement.

**NEW & NOTEWORTHY** Our results reveal that interacting humans inconspicuously modulate muscle activation to extract accurate information about the common target while considering their own and the partner’s sensorimotor noise. A novel computational model was developed to decipher the underlying mechanism: muscle coactivation is adapted to combine haptic information from the interaction with the partner and own visual information in a stochastically optimal manner. This improves the prediction of the target position with minimal metabolic cost in each partner, resulting in the lead of the partner with the most accurate visual information.

## Graphical abstract

**Figure:**
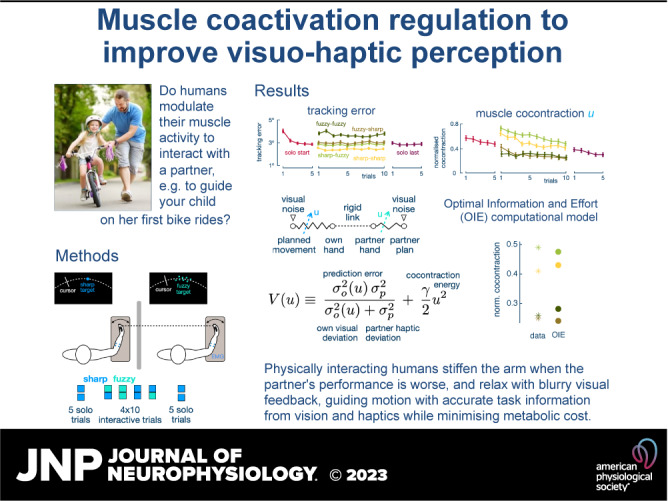

## INTRODUCTION

**Figure 1.**
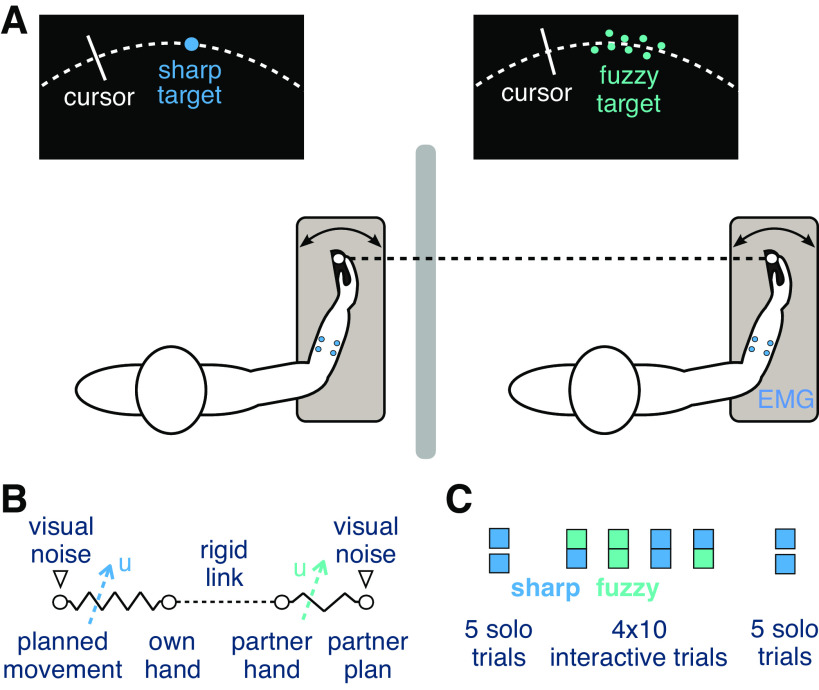
Experiment to study how muscle coactivation varies during the joint tracking of 2 connected humans. *A*: the partners track the same randomly moving target with their wrist flexion-extension movement while being connected with a rigid virtual bar. Their wrist flexion/extension movement is recorded, as well as the myoelectrical activity of a flexor-extensor muscle pair. EMG, electromyogram. *B*: diagram of mechanical interaction with the partner and with own movement plan. The interaction with the partner’s hand depends on the stiffness of the connection to their motion plan modulated by their coactivation *u*. Both own and partner movement plans are affected by the respective visual noise. *C*: protocol of the experiment to study the effect of visual noise on either partner’s performance and coactivation.

Human muscles are elastic elements that increase stiffness and shorten with activation (1). The central nervous system (CNS) regulates the limbs’ stiffness by coordinating muscle activation to shape the interaction with the environment (2, 3), but how this affects haptic sensing is not known. When two connected individuals carry out a task together (Fig. 1*A*), they exchange haptic information about their motion plan to combine with own visual information and improve their accuracy (4). Critically, haptic information transferred by the mechanical connection is modulated by their muscle coactivation (Fig. 1*B*). Could individuals regulate their muscles’ activation to adapt the limbs’ stiffness and better sense the partner’s movement?

To understand how physically connected individuals control their arm coactivation, we observed 22 pairs of subjects or *dyads* tracking a common randomly moving target using wrist flexion and extension (Ref. 5, Fig. 1*A*). Studies on the adaptation to unpredictable force fields (6, 7) suggest that muscle coactivation would increase with the magnitude of error to their motion plan (3) independent of its source. However, we hypothesized that interacting humans can adapt their muscle coactivation to their own sensorimotor noise and to haptic noise resulting from the interaction with the partner.

To test this hypothesis, we carried out an experiment in which the visual feedback provided to the partners was manipulated. The target observed by each partner on their individual monitor was either *sharp* (a 8-mm large disk) or *fuzzy* (a dynamic cloud of 8 normally distributed dots). We analyzed the tracking performance and wrist muscle activation of each partner and developed a computational model to understand how muscle activation is adapted to each specific noise condition.

## METHODS

### Participants

The experiment was approved by the Joint Research Compliance Office at Imperial College London. Forty-four participants without known sensorimotor impairments aged 18–37 yr, including 16 females, were recruited. Each participant gave written informed consent before participation. Thirty-seven participants were right-handed, five left-handed, and two ambidextrous, as assessed with the Edinburgh Handedness Inventory (8). The participants carried out the experiment in pairs or dyads. To avoid sex-related effects on the interaction behavior (9), the experiment was carried out by same-sex dyads.

### Experimental Setup

The two partners of each dyad were seated on height-adjustable chairs, next to the Hi5 dual robotic interface (10). They held their respective handle with the wrist of the dominant hand and received visual feedback of the flexion/extension movement on a personal monitor (Fig. 1*A*). No visual feedback of the partner’s position was available as the two participants were separated by a curtain, and they were instructed not to speak to each other during the experiment.

Each Hi5 handle is connected to a current-controlled DC motor (MSS8; Mavilor) that can exert torques of up to 15 Nm and is equipped with a differential encoder (RI 58-O; Hengstler) to measure the wrist angle and a sensor (TRT-100; Transducer Technologies) to measure the exerted torque in the range [0,11.29] Nm. The two handles were controlled at 1 kHz with LabVIEW Real-Time v14.0 (National Instruments) and a data acquisition board (DAQ-PCI-6221; National Instruments) while the data were recorded at 100 Hz.

The activation of two antagonist wrist muscles, the flexor carpi radialis (FCR) and extensor carpi radialis longus (ECRL), was recorded during the movement from each participant. Electromyographic (EMG) signals were measured with surface electrodes using the medically certified noninvasive 16-channel EMG system (10). The EMG data were recorded at 100 Hz.

### Tracking Task

The two partners were required to track the same *visual target* “as accurately as possible” on their respective monitor with (in degrees)

(*1*) $$\begin{array}{l} q^{*}(t)\equiv 18.5\;\sin\left(2.031\;t^{*}\right)\;\text{sin}\left(1.093\;t^{*}\right)\\ t^{*}\equiv t+t_{0},\;0\leq t\leq 20\;s \end{array}$$

using flexion-extension movements (where *t* is the time). To prevent the participants from memorizing the target’s motion, t* started in each trial from a randomly selected offset time {*t*_0_ ∈ [0, 20]s|q*(*t*_0_) ≡ 0} of the multisine function. The respective *tracking error*

(*2*) $$e\equiv {\left(\frac{1}{T}\int_{0}^{T}{\left[q^{*}(t)-q(t)\right]}^{2}dt\right)}^{\frac{1}{2}},\;T\equiv 20\;s$$

was displayed at the end of each 20-s-long trial.

After each trial, the target disappeared and the participants had to place their respective cursor on the starting position at the center of the screen. The next trial then started after a 5-s rest period and a 3-s countdown. The initialization of the next trial started when both participants placed their wrist on the starting position, so that each participant could take a break at will between trials by keeping the cursor away from the center of the screen.

### Experimental Conditions and Protocol

In *solo trials*, the two partners moved the wrist independently of each other. In *interactive trials*, the partners’ wrists were connected by a stiff virtual spring with torque (in Nm)

(*3*) $$\tau (t)=\;0.30\;[q_{p}(t)-q_{o}(t)]\;$$

where *q*_o_ and *q*_p_ (in degrees) denote own and partner’s wrist angles, respectively.

The interaction trials were carried out under two different visual feedback conditions. In the *sharp condition* the target was displayed as an 8-mm-diameter disk. In the *fuzzy condition* the target trajectory was displayed with eight normally distributed dots around the target. The cloud of dots was defined by three parameters, randomly picked from independent Gaussian distributions: the vertical distance to the target position η ∈ N(0, 15 mm), the angular distance to the target position η*_q_* ∈ N(0, 4.58°), and the angular velocity η*_q_* ∈ N(0, 4.01°/s). Each of the eight dots was sequentially replaced every 100 ms.

A calibration of the measured EMG (described in *Muscle Activation Calibration and Cocontraction Calculation*) was first carried out to map the raw EMG signal (in mV) to a corresponding torque value (in Nm), so that the activity of each participant’s flexor and extensors can be compared and combined in the data analysis. After this calibration, the participants carried out five initial solo trials to learn the tracking task and the dynamics of the wrist interface. This was followed by four blocks of 10 interaction trials, each with one of the four different noise conditions [fuzzy(self)-sharp(partner): FS, SF, SS, FF] presented in a random order, followed by five control solo trials (Fig. 1*C*). The participants were informed when an experimental condition would be changed but not that they were connected to the partner or which condition they would be encountering in the next trials.

### Muscle Activation Calibration and Cocontraction Calculation

The participants placed their wrist in the most comfortable middle posture, set to 0°. Constrained at that posture, they were then instructed to sequentially flex or extend the wrist to exert torque. Each phase was 4 s long and was followed by a 5-s rest period to avoid fatigue. The latter period was used as a reference activity in the relaxed condition. This procedure was repeated four times at flexion/extension torque levels of {1,2,3,4} Nm and {−1,−2,−3,−4} Nm, respectively.

The recorded muscle activity of each participant was then linearly regressed against the torque values. The raw EMG signal was *1*) high-pass filtered at 20 Hz by using a second-order Butterworth filter to remove drifts in the EMG and *2*) rectified and passed through a low-pass second-order Butterworth filter with a 5-Hz cutoff frequency to obtain the envelope of the EMG activity.

The torque of the flexor muscle could then be modeled from the envelope of the EMG activity *u*_f_ as

(*4*) $$\begin{array}{l} \tau_{f}(t)=\alpha_{0}\;u_{f}(t)+\alpha_{1}\;,\;\alpha_{0},\alpha_{1}>0 \end{array}$$

and similarly for the torque of the extensor muscle τ_e_. The torque due to *reciprocal activation* of the FCR [with τ_f_(*t*) ≥ 0] and ECRL [τ_e_(*t*) ≤ 0] was computed as

(*5*) $$v(t)\equiv \tau_{f}(t)+\tau_{e}(t)$$

and the torque due to *muscle coactivation* as

(*6*) $$u(t)\equiv \text{min}\{\tau_{f}(t),|\tau_{e}(t)|\}$$

**Figure 2.**
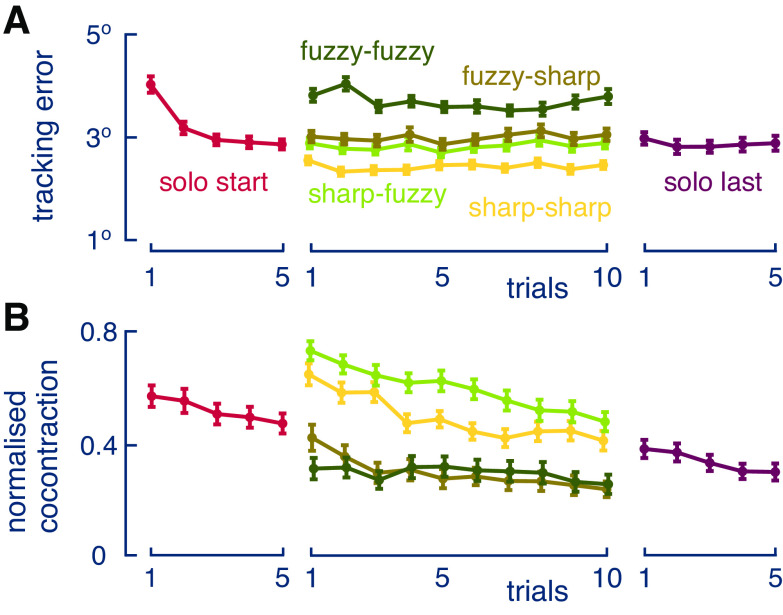
*A*: evolution of group mean tracking error charted as a function of trials, where error bars represent 1 SE. The error saturated in the initial solo trials and increased with visual and haptic noise. *B*: evolution of normalized cocontraction as a function of trials, where error bars represent 1 SE.

The average coactivation over all participants (as shown in Fig. 2) was computed from each participant’s normalized coactivation

(*7*) $$u_{n}\equiv \frac{\bar{u}-{\bar{u}}_{\min}}{{\bar{u}}_{\max}-{\bar{u}}_{\min}}\;,\;\bar{u}\equiv \frac{1}{T}\int_{0}^{T}u(t)dt,\;T\equiv 20\;s$$

with ${\bar{u}}_{\min}$ and ${\bar{u}}_{\max}$ the minimum and maximum of the means of all trials of the specific participant.

## EXPERIMENTAL RESULTS

To evaluate the short-term adaptation within each condition, analysis was carried out on the average measurements from the first and second half of trials (epochs). As the tracking error and muscle cocontraction were influenced by both the subject’s own visual noise and the partner’s visual noise (perceived through the spring interaction as haptic noise), they were analyzed individually with a three-way repeated-measures ANOVA with visual noise, haptic noise, and epoch as the factors in the analysis of the tracking error and cocontraction level. Bonferroni–Holm correction was used to correct for type I error in multiple post hoc comparisons for each metric.

We see in Fig. 2*A* that the error had decreased by the last of the initial solo trials to the same degree as the average of the last solo trials (*P* = 0.27, paired-samples Wilcoxon test). This indicates stable performance to analyze the different interaction conditions. The ANOVA of tracking error in these conditions indicated that the magnitude of the tracking error depended on both visual noise level [*F*(1,43) = 359.95, *P* < 0.001, $\eta_{p}^{2}$ = 0.21] and haptic noise level [*F*(1,43) = 210.46, *P* < 0.001, $\eta_{p}^{2}$ = 0.12]. There was an interaction effect between visual and haptic noise [*F*(1,43) = 14.83, *P* < 0.001, $\eta_{p}^{2}$ = 0.008]. Post hoc comparisons showed that there was no significant difference between noise conditions SF and FS (*P* = 0.14), while both were greater than in the SS condition (*P* < 0.001) and smaller than in the FF condition (*P* < 0.001). The tracking error remained at a similar level between the first and the second epochs (*P* = 0.38), and there was no interaction effect between either noise level and epoch (*P* > 0.15). Note that the connection with the partner improved the tracking performance, so that the error was smaller in the SS condition than in the solo condition [paired *t* test, *t*(43) = 6.22, *P* < 0.001]. This confirms observations in a similar task carried out on different setups (4, 11, 12).

The cocontraction level decreased with the epoch [*F*(1,43) = 53.58, *P* < 0.0005, $\eta_{p}^{2}$ = 0.56], similar to what was observed during the learning of force fields (3, 13) (Fig. 2*B*). Importantly, the cocontraction decreased with a larger level of own visual noise [*F*(1,43) = 85.91, *P* < 0.0005, $\eta_{p}^{2}$ = 0.67], whereas it increased with haptic noise from the interaction with the partner [*F*(1,43) = 5.53, *P* < 0.03, $\eta_{p}^{2}$ = 0.11]. Post hoc comparisons confirmed that all differences between the combinations of the visual and haptic noises were significant, with the exception of FS versus FF (*P* = 0.99).

**Figure 3.**
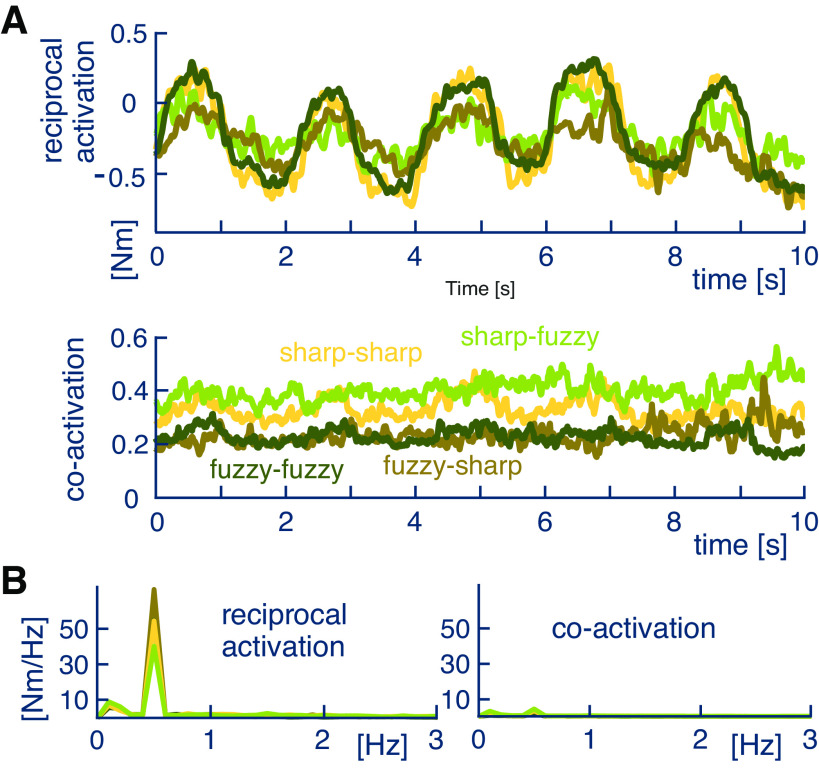
Activation of antagonist wrist muscles in the last trial. *A*: reciprocal activation (RA) and coactivation (CA) in the last trial averaged over subjects. The waveforms were aligned in time before averaging to compensate for the different temporal delays in *Eq. 1*. Selecting the first 10 s in aligned waveform enables us to consider 25 of the 44 subjects for the averaging. *B*: the RA spectrum exhibits the same peaks as the target movement, whereas the CA spectrum is essentially flat.

To analyze how this adaptation is affected by the wrist antagonist muscles, we computed their reciprocal activation (RA, defined in *Eq. 5*) and coactivation (CA, *Eq. 6*) in the last trial. As shown in Fig. 3*A*, the mean RA over the subjects varied (with standard deviation 0.5 Nm) much more than the CA (standard deviation 0.1 Nm). Furthermore, RA was well correlated with the hand trajectory (|Pearson correlation| > 0.8 with *P* < 0.05), but CA was not correlated (|correlation| < 0.1 with *P* < 0.05). Figure 3*B* shows that RA exhibited a spectrum similar to the target trajectory, with peaks around 0.15 Hz and 0.5 Hz, whereas the spectrum of CA was essentially flat, exhibiting only a much smaller peak around 0.5 Hz. These results show that the RA was driving the tracking movement whereas the level of CA was regulated specifically to each noise condition.

In summary, the experimental results indicate that during interaction the CNS spontaneously regulated muscle coactivation considering the level of the visual noise on one’s own and the partner’s targets, in agreement with our hypothesis. As a consequence, the partner with more accurate visual information increases their coactivation and thus their stiffness (14) and leads the movement. Conversely, the partner with less accurate visual information decreases their stiffness and thus their influence on the dyad’s motion control. The interactive behavior of the dyad results from the equilibrium of the coactivation adaptation in the two partners modulated by their respective sensory noise.

## COMPUTATIONAL MODELING

What is the principle behind this adaptation? As the participants were not aware of the connection with the human partner (11), would coactivation be adapted as when interacting with a dynamic environment? Therefore we first tested the computational model of Ref. 3 that explains the motor learning in novel force fields. In this model, the coactivation *u* increases with each new trial to minimize tracking error *e* and decreases to minimize effort, according to

(*8*) $$u_{\mathrm{new}}\equiv \alpha e+(1-\gamma )\;u\;,\;0<\alpha ,\;0<\gamma <1$$

For each of the four noise conditions *ij* ∈ {SS, SF, FS, FF}, the initial cocontraction level $\left\{{\hat{u}}_{ij}(1)\right\}$ was set as the initial experimental value $\left\{u_{ij}(1)\right\}$. Then, by using the respective trial-by-trial tracking error {*e_ij_*(*k*)}, *k* = 1,…, 10 from the experiment, the adaptation parameters α,γ in the computational model of *Eq. 8* were computed to minimize the error between the learned cocontraction values after 9 iterations $\left\{{\hat{u}}_{ij}(10)\right\}$ and the corresponding data $\left\{u_{ij}(10)\right\}$ in last experiment’s trial:

(*9*) $$\left(\alpha^{*},\gamma^{*})\equiv \;\underset{\alpha ,\gamma }{\arg\;\min\;}\{\sum_{i,j}{\left[{\hat{u}}_{ij}(10)-u_{ij}(10)\right]}^{2}\right\}$$

The parameters α* ≡ 0.5, γ* ≡ 0.06 were found by using a grid search with a step 0.01 in the range [0,2] × [0,1.5].

**Figure 4.**
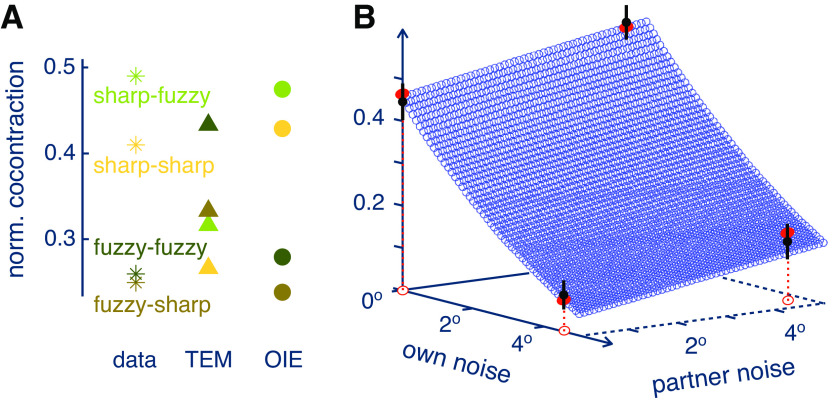
Results of computational modeling of the coactivation adaptation to own and partner noise. *A*: comparison of coactivation observed during the experiment and predicted by the 2 models described in the text. The tracking error minimization (TEM) model cannot catch the modulation of coactivation with different noise conditions observed in the data, whereas the optimal information and effort (OIE) model predicts their trend well. *B*: the OIE model predicts a decrease of coactivation with own noise and an increase with partner noise.

Simulation of the learning during the 10 trials of each condition with this *tracking error minimization* (TEM) model predicted cocontraction at a level increasing with the corresponding tracking error (Fig. 4*A*). This prediction is qualitatively different from the data, such as larger coactivation in the fuzzy relative to the sharp visual feedback condition (e.g., compare the FF and SS conditions in Fig. 4*A*). Therefore, the TEM model cannot explain the adaptation in the coactivation during interaction.

The hand movement depends on the guidance toward the planned movement and on the connection to the partner (Fig. 1*B*). As the stiffness of the guidance increases with own coactivation (2), it is possible to weigh these two influences. Coactivation should decrease to lower the guidance to the planned movement when it is affected by visual noise. Conversely, the guidance to the planned motion should increase to counteract the effect of haptic noise when the partner receives noisy visual feedback. Therefore, the coactivation may depend both on the statistical information determining the quality of the planned motion, which relies primarily on vision, and on the partner’s accuracy in tracking the common target.

We thus propose that coactivation is modulated to maximize information from visual information and haptic information from the interaction with the partner. We introduce the *optimal information and effort* (OIE) model that addresses the trade-off between motion guidance and interaction noise attenuation, by selecting coactivation *u* to minimize the *prediction error E*(*u*) and *metabolic cost u*^2^, with the cost function

(*10*) $$V(u)=E(u)+\frac{\gamma }{2}u^{2}\;,\;E(u)\equiv \frac{\sigma_{o}^{2}(u)\;\sigma_{p}^{2}}{\sigma_{o}^{2}(u)+\sigma_{p}^{2}}$$

where σ_o_(*u*) results from the effect of own visual noise on the arm movement and σ_p_ from the interaction with the partner. This minimization can be carried out through gradient descent minimization:

(*11*) $$\begin{array}{l} u_{\mathrm{new}}=u-\frac{dV(u)}{du}=-\frac{dE(u)}{du}+(1-\gamma )\;u\;,\;0<\gamma <1\\ -\frac{dE(u)}{du}={\left[\frac{\sigma_{p}^{2}}{\sigma_{o}^{2}(u)+\sigma_{p}^{2}}\right]}^{2}\left[-\frac{d\sigma_{o}^{2}(u)}{du}\right]>0 \end{array}$$

The target tracking arises from the internal guidance to the planned motion and the mechanical connection with the partner, both being subjected to the noise in the respective visual feedback (Fig. 1*B*).

How should the deviation σ_o_ be modeled? First, let σ_νo_ describe the tracking deviation of own wrist movement due to the target cloud. Second, the wrist’s compliance influences the tracking performance and brings in noise in the planned movement (5). Assuming that these two effects are independent and that the wrist’s viscoelasticity results in zero mean noise with deviation σ_κo_(*u*), the deviation in the wrist can be calculated as

(*12*) $$\sigma_{o}^{2}(u)=\;\sigma_{vo}^{2}+\sigma_{\kappa o}^{2}(u)$$

where σ_κo_(*u*) = 5.18 + 49.65*e*^−6.11^*^u^* was identified as the least-square fit of data from the haptic experiment in Ref. 15.

Considering the relationship between the deviation σ_κo_ and the wrist’s viscoelasticity, the visual noise deviation and the partner’s noise deviation each have two values, resulting in four parameters to identify: $\left\{\sigma_{vo}^{s},\sigma_{vo}^{f},\sigma_{p}^{s},\sigma_{p}^{f}\right\}$, where “s” corresponds to the sharp and “f” to the cloudy target. These parameters, used in the noise models of *Eq. 12*, were computed by minimizing the variation of the cost derivative:

(*13*) $$\begin{array}{l} \left(\sigma_{vo}^{s*},\sigma_{vo}^{f*},\sigma_{p}^{s*},\sigma_{p}^{f*}\right)\equiv \\ \underset{\sigma_{\nu o}^{s},\sigma_{vo}^{f},\sigma_{p}^{s},\sigma_{p}^{f}}{\arg\;\min}\{\sum_{i}\sum_{j}{\left[\frac{\partial V}{\partial u}\left(u_{ij}(10),\;\sigma_{vo}^{\left(i\right)},\;\sigma_{p}^{\left(j\right)}\right)\right]}^{2}\} \end{array}$$

Using the collected cocontraction data {*u_ij_*(10)}, a grid search for $\left(\sigma_{vo}^{s},\sigma_{vo}^{f},\sigma_{p}^{s},\sigma_{p}^{f}\right)$ with manually presearched range in [0,12] × [0,20] × [0,10] × [0,20] with step 0.2 yields $\sigma_{vo}^{s*}=10,\;\sigma_{vo}^{f*}=18.8,\;\sigma_{p}^{s*}=5.2,\;\sigma_{p}^{f*}=6$, where for each grid point γ* = 0.65 was the solution of

(*14*) $$0\equiv \frac{d}{d\gamma }\left(\sum_{i}\sum_{j}{\left[\frac{\partial V}{\partial u}(u_{ij}(10),\sigma_{vo}^{\left(i\right)},\sigma_{p}^{\left(j\right)})\right]}^{2}\right)$$

As can be seen in Fig. 4*A*, the OIE model predicts the modulation of coactivation with both own visual noise and haptic noise from the partner as observed in the data, in contrast to the TEM model. The adequacy of the OIE is further shown from the Akaike information criterion (AIC): the small sample size-normalized AIC value (16, 17) using OIE model prediction is −6.2, smaller than the value of −2.8 for the TEM model, suggesting that OIE is a better model than TEM considering the information loss and the number of independent parameters. The OIE model can be used to predict the coactivation for any level of own and partner’s noise, as shown in Fig. 4*B*.

## DISCUSSION

These experimental and computational results demonstrate that interacting humans modulate their muscles’ activation to extract accurate information about the common target considering own and partner’s noise. Although it has been known that muscles’ activation is adapted to shape the mechanical interaction with the environment (2, 7), our results reveal that the CNS further regulates the limbs’ viscoelasticity to extract optimal sensory information from the interaction. Not only do individuals share haptic information to extract each other’s motion plan (4), but they further learn muscle activation to improve this estimation.

These results could not be explained by previous models of coactivation adaptation in dynamic environments, which consider only the error in the task performance (3, 18, 19). However, the observed coactivation changes with both own and partner’s noise were well predicted by the OIE model introduced in this article. The OIE adapts coactivation to maximize information from vision and haptics arising from the interaction with the partner while minimizing energy by reducing coactivation.

This mechanism skillfully regulates coactivation to extract maximum sensory information while exploiting the guidance potential from the partner: Coactivation decreases to rely more on the partner guidance when vision is fuzzy and increases when the interaction with the partner is noisy. As end-point stiffness increases with the coactivation (14), the partner with more accurate information appears to lead the movement. This “leadership” does not depend on any partner’s character but relies on the quality of sensory information in the partners. The interactive behavior then results from the concurrent adaptation of muscle activation in the partners induced by their respective sensory noise, where the more skilled partner increases their coactivation and thus their lead and the less skilled partner decreases coactivation and thus their influence on the dyad’s motion control. While these results were observed in a collaborative task, dyads with conflicting goals may use more complex strategies.

The OIE model, specifying how the CNS adapts coactivation to minimize prediction error and energy, extends previous work on motor learning and adaptation. Although the models in Refs. 20, 21 could determine the motion kinematics in the next trial from the movement error in previous trials, this new model also considers the limbs’ neuromechanics and can thus predict the interaction force and the subsequent muscle activity during motion. The OIE also extends optimal and nonlinear adaptive control models (3, 19, 22–24) by considering the consequence of action on the acquired sensory information from the environment, closing the loop between the sensory and motor actions. This adaptive sensing mechanism may give rise to interactive robots that can modify their rigidity to optimally sense their user and best assist them.

## DATA AVAILABILITY

Data will be made available upon reasonable request.

## GRANTS

This work was supported in part by the EC H2020 Grants PH-CODING (FETOPEN 829186), CONBOTS (ICT 871803), and REHYB (ICT 871767).

## DISCLOSURES

No conflicts of interest, financial or otherwise, are declared by the authors.

## AUTHOR CONTRIBUTIONS

H.B., G.C., S.H., S.E. and E.B. conceived and designed research; H.B. performed experiments; H.B., G.C., X.C., A.T., S.E., and E.B. analyzed data; H.B., G.C., X.C., A.T., S.H., and E.B. interpreted results of experiments; H.B., G.C., X.C., and E.B. developed the computational modeling; H.B., X.C., and E.B. prepared figures; G.C., X.C., S.E., and E.B. drafted manuscript; H.B., G.C., X.C., A.T., S.H., S.E., and E.B. edited and revised manuscript; H.B., G.C., X.C., A.T., S.H., S.E., and E.B. approved final version of manuscript.
